# Scrofuloderma and Miliary Tuberculosis in a 27-Year-Old Nurse

**DOI:** 10.7759/cureus.84061

**Published:** 2025-05-13

**Authors:** Francisco Javier Lugo Rincón-Gallardo, Néstor Daniel Rodríguez Trujillo, Azalea Guadalupe Altamirano De La Cruz, Alejandra Priscila Castillo Gómez, Karen S Arrazola

**Affiliations:** 1 Internal Medicine, General Hospital of the Institute of Social Security and Services for State Workers, Querétaro, MEX; 2 Internal Medicine, Hospital General Juan María de Salvatierra, La Paz, MEX; 3 Internal Medicine, Hospital San Javier, Guadalajara, MEX

**Keywords:** clinical dermatology, internal medicine specialist, pulmonary tuberculosis, scrofuloderma, skin infections, tuberculosis

## Abstract

A previously healthy 26-year-old female nurse presented with fever, diaphoresis, and occasional cough for over two months. She visited the emergency department several times and never had a correct diagnosis. Eventually, she presented to the emergency department of the ISSSTE General Hospital of Querétaro and was admitted to the Internal Medicine service. On physical examination, she had a non-painful, mobile, well-defined, and indurated nodule in the left cervical region, approximately 20 mm in diameter. The skin had erythematous-violaceous discoloration on top of the nodule with two central ulcerations and discrete seropurulent discharge when applying pressure. The nodule was biopsied. Histologic report identified caseous necrosis, and, simultaneously, chest X-ray and chest CT scan findings of miliary tuberculosis. Subsequently, polymerase chain reaction identification of *Mycobacterium tuberculosis* confirmed the diagnosis of lymph node tuberculosis with contiguous scrofuloderma and pulmonary miliary tuberculosis. After 12 months of treatment, clinical recovery was accomplished.

## Introduction

Scrofuloderma, also known as tuberculosis colliquativa cutis, began to be mentioned in medical literature around 1880. The initial term that described this disease was scrofulous gumma, introduced by Ernst Besnier in 1883. The Latin meaning of scrofa is pig due to the resemblance of a pig and the affected individual, with thickened tissue around the neck. Gumma was added because of the nodules’ resemblance to the syphilitic gummas. The discovery of *Mycobacterium tuberculosis *bacilli by Robert Koch in 1882 changed how humanity practiced medicine, and the association of scrofulous gumma with the bacilli was eventually discovered [1].

Scrofuloderma is the most common skin tuberculosis manifestation in Mexico, responsible for 51% of all dermatological infections. It affects all age groups, but mostly children and young people, often associated with malnutrition. Scrofuloderma is a skin lesion caused by the direct extension of the underlying *M. tuberculosis* complex infection (*M. tuberculosis*, *M. bovis*, *M. africanum*, *M. microti*, *M. canetti*, and *M. innipedii*). *M. tuberculosis* is the predominant strain (95%), with other strains documented in only 5% of the scrofuloderma cases. In scrofuloderma, primary tuberculosis infection is usually located in the lymph nodes, joints, or bones, with coexisting lung infection in 35% of the cases (associated with poor immunity), which contiguously spreads to the skin that covers the inflamed primary site of the infection. The affected skin can turn indurated, purplish, and ulcerate with crusty, irregular pale granulomatous tissue. Usually, the distribution of affection involves the neck region, axilla, or groin due to the lymph node conglomeration in the mentioned sites. The classic clinical presentation is an asymptomatic, well-defined, firm, movable, and non-painful nodule, sometimes erythematous, that ulcerates over time with discharging pus from the apertures or sinus tract formation. Scar formation is common, and most of the scarring is keloid or retractile. Skin lesions evolve slowly, and other symptoms may accompany the cutaneous lesions, such as fever and weight loss [1,2]. The gold-standard diagnosis test is the culture of the skin lesion, revealing *M. tuberculosis* bacteria. Another study that helps clinicians raise the suspicion of a mycobacterial infection is a positive interferon-gamma release assay. Skin biopsy can demonstrate caseating epithelioid granulomas that contain acid-fast bacilli, which appear as red bacilli with the Ziehl-Neelsen stain. Scrofuloderma may heal even without treatment, but it takes years to do so and can result in extensive scarring. Differential diagnosis of scrofuloderma should be ruled out, particularly hidradenitis suppurativa, atypical mycobacterial infections, sporotrichosis, actinomycosis, tumor metastasis, and bacterial abscesses.

## Case presentation

A 26-year-old Mexican female nurse with no prior medical history suddenly developed insidious evening fevers up to 39°C, profuse diaphoresis, occasional white sputum-producing cough, asthenia, adynamia, arthralgias, and weight loss of about 2 kg over the last two months. Despite multiple antibiotic treatments and visits to numerous emergency departments, no improvement was accomplished. She was eventually admitted to the ISSSTE General Hospital in Querétaro in the Internal Medicine department.

**Figure 1:**
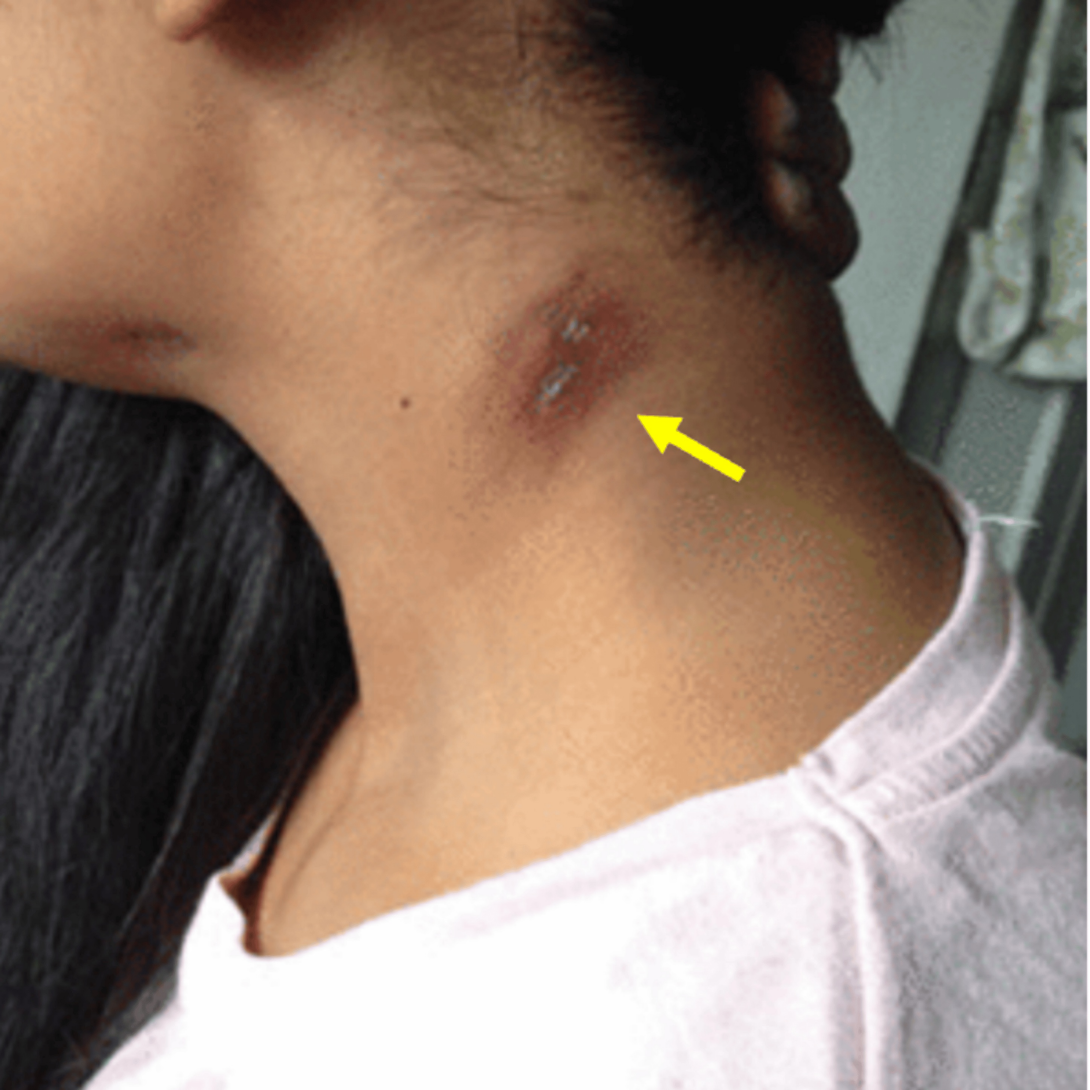
Cutaneous nodule with overlying hyperchromatic maculae and two central openings.

On clinical examination, a well-defined, indurated, non-painful, and non-mobile nodule, approximately 6 mm in diameter, with two central ulcers and discrete seropurulent discharge when applying pressure, was seen on the left side of the neck (Figure 1). The surrounding skin had erythematous-violaceous discoloration. Initial laboratory findings are shown in Table 1.

**Table 1:** Initial laboratory tests.

Initial laboratory tests	Patient results	Reference range
Hemoglobin	14.3 g/dL	12.0–16.0 g/dL
Platelets	276,000/µL	145,000–450,000/µL
Leukocytes	7,000/µL	4,500–11,000/µL
Neutrophils	4,900/µL	2,500–8,000/µL
Lymphocytes	1,700/µL	1,500–4,500/µL
Lactate dehydrogenase	636 IU/L	140–280 IU/L
Aspartate aminotransferase	219 U/L	2–40 U/L
Alanine aminotransferase	136 U/L	0–45 U/L
Carcinoembryonic antigen	3.2 ng/dL	<5 ng/dL
Alpha-fetoprotein	4.1 ng/dL	0–20 ng/dL
Polymerase chain reaction detection of HIV	Not detected	Not detected
Polymerase chain reaction detection of hepatitis B virus	Not detected	Not detected
Polymerase chain reaction detection of hepatitis C virus	Not detected	Not detected
Cytomegalovirus IgM and IgG	IgM (-), IgG (-)	Positive/Negative
Herpes simplex IgM and IgG	IgM (+), IgG (-)	Positive/Negative
Toxoplasma IgM and IgG	IgM (-), IgG (-)	Positive/Negative
Rubella	IgM (-), IgG (-)	Positive/Negative
Rheumatoid factor	29 IU/mL	0–20 IU/mL
Antinuclear antibodies	1:40	Positive/Negative

**Figure 2:**
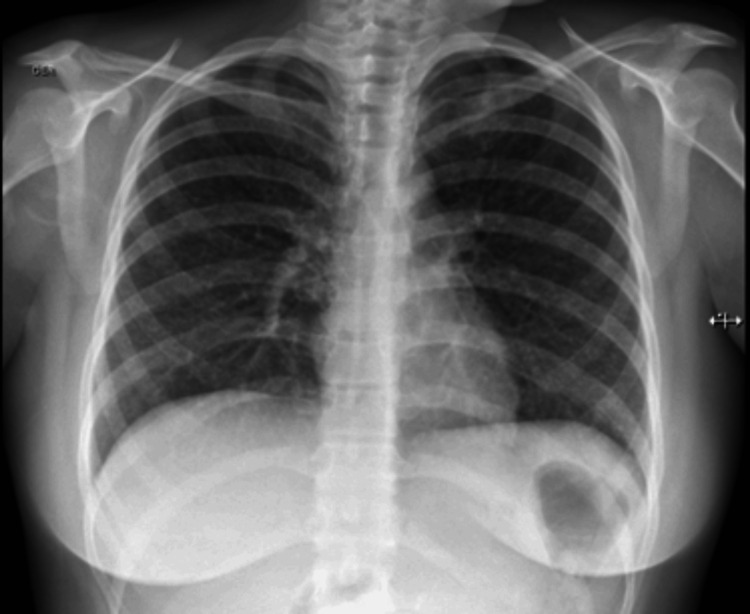
Chest X-ray showing diffuse micronodular radiopacities on both lungs.

**Figure 3:**
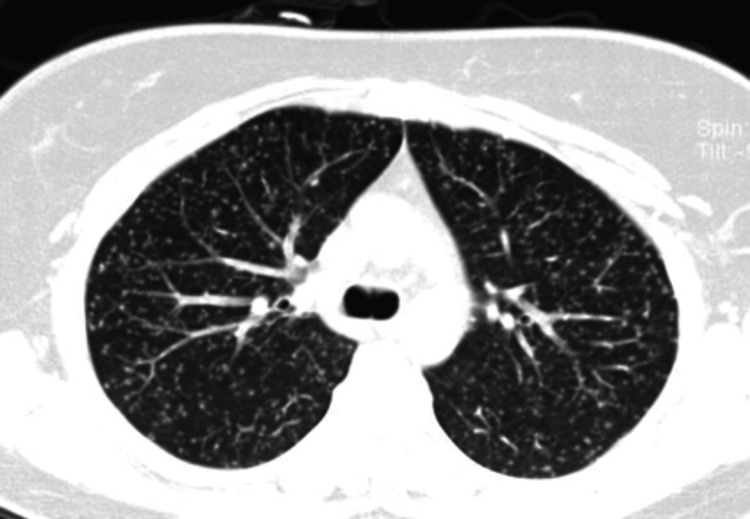
CT scan showing the diffuse micronodular hyperdensities also known as miliary findings of tuberculosis.

Due to suspicion of *M. tuberculosis* infection, a chest X-ray was requested, which revealed small nodular radiopacities distributed diffusely and uniformly in both lungs (Figure 2). The radiopacities were located predominantly in the lower lobes. Concerns about miliary tuberculosis led to a thoracoabdominal CT scan, showing a diffuse, bilateral micronodular lung pattern with a budding tree sign, along with para-aortic lymph node clusters (Figure 3).

**Figure 4:**
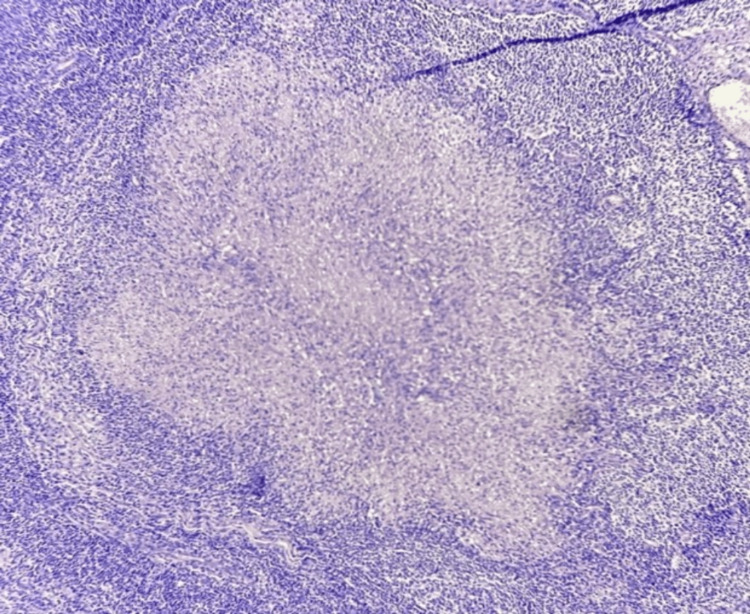
Hematoxylin and eosin staining of the lymph node with central caseous necrosis.

**Figure 5:**
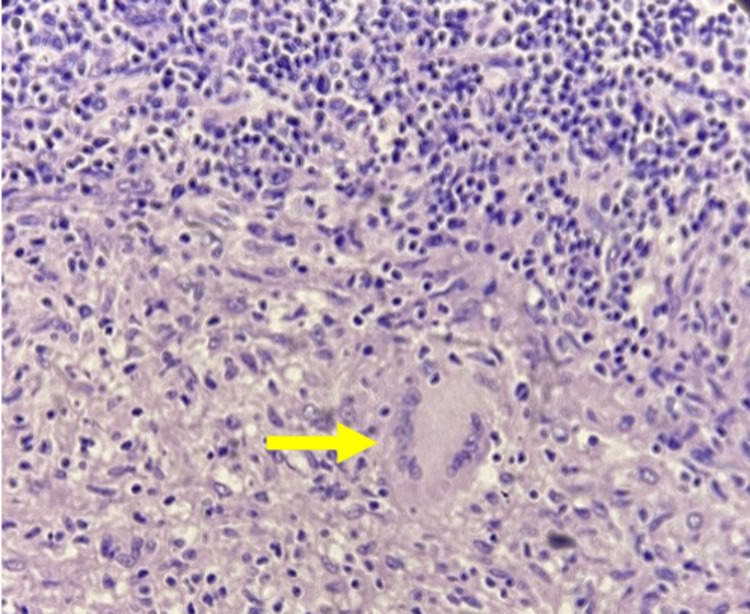
Langerhans cells with epithelioid cells on macrophagic conversion and superior peripheral lymphocytic band.

At the same time, a cervical ultrasound was requested, revealing an ovoid, hypoechoic, lobulated morphology in the left dorsal neck region, measuring 10.9 × 7.0 cm, located 5.0 mm deep from the epidermis. There was minimal vascularity upon Doppler examination. Consequently, an excisional biopsy of the left cervical lymph node was performed, indicating chronic granulomatous lymphadenopathy, with well-circumscribed granulomas containing multinucleated giant cells and caseous necrosis, with caseous necrosis and dense granulomatous inflammatory infiltrate in the reticular dermis, consistent with tuberculosis (Figures 4, 5).

Ziehl-Neelsen staining for acid-fast bacilli was not available in the hospital. Later, a QuantiFERON test was positive, and a polymerase chain reaction (PCR) confirmed the presence of *M. tuberculosis*, leading to the diagnosis of peripheral ganglionar tuberculosis with contiguous scrofuloderma and miliary tuberculosis.

Antituberculosis treatment was initiated (rifampicin 600 mg, pyrazinamide 1500 mg, ethambutol 1,200 mg, and isoniazid 300 mg; intensive phase; four tablets of the combination from Monday to Saturday) for two months with a favorable response. Subsequently, isoniazid 800 mg and rifampicin 600 mg (continuation phase; two tablets of the combination on Monday, Wednesday, and Friday) were continued until the completion of 12 months of treatment. At the sixth-month follow-up, reassessment showed a favorable response with the presence of hypertrophic scarring in the left supraclavicular area secondary to the cutaneous process.

A one-year radiological follow-up revealed a significant decrease in nodular radiopacities on chest X-ray, despite remaining diffusely and uniformly distributed in both lungs. The chest CT control exhibited a notable reduction compared to the initial scan in the diffuse, bilateral, random micronodular lung pattern.

## Discussion

Tuberculosis is a preventable infectious disease with one of the highest morbidity and mortality rates among other contagious microorganisms because the infection can be systemic and potentially affect any organ, including the skin [3]. Pulmonary infection by *M. tuberculosis* accounts for 80% of the infections globally, of which 8% are miliary or disseminated tuberculosis. Extrapulmonary infection is responsible for only 8.4-13.7% of the global tuberculosis infections, of which only 0.5-2% affect the skin [3,4].

Scrofuloderma, also known as tuberculosis colliquative cutis, is the predominant type of skin tuberculosis in underdeveloped countries and immunodeficient patients. It affects all age groups, but predominantly affects children, adolescents, and the elderly [5].

The clinical presentation usually manifests as multiple or unique deep nodules, as it evolves, changes in the underlying skin can manifest, characterized by erythematous, purplish, or brown discoloration. Over time, the nodule begins to form a cold abscess at its center, which eventually softens and ulcerates, with discharge of caseous and purulent material through its aperture or sinus tract formation. Without treatment, scrofuloderma may heal spontaneously over months and years, leaving behind bridging or cerebriform scars, as well as pockets of retraction [6,7].

Of the people suffering from skin tuberculosis, 33% have been shown to have miliary tuberculosis at the same time. Miliary tuberculosis is defined as the hematogenous spread of *Mycobacteria*, affecting the lung and other organs. The high correlation of miliary and skin tuberculosis is due to the high bacterial load in miliary tuberculosis, which spreads through the bloodstream to any other organ. Once primary tuberculosis is settled, the infected tissue can easily evolve to contiguously spread directly to the overlying cutaneous surface and cause scrofuloderma [8]. Cervical lymph nodes are the most common underlying primary infection that develops into scrofuloderma [9].

The classification of cutaneous tuberculosis is based on the three routes of infection, i.e., exogenous, endogenous, and hematogenous/lymphatic dissemination. Scrofuloderma is classified as endogenous tuberculosis because it is caused by a contiguous spread from an underlying primary tuberculosis focus, such as bone, joint, testicles, epididymis, or lymph nodes, that evolves to a secondary contiguous spread to the skin [5,6]. According to literature, up to 72% of patients with peripheral ganglionar tuberculosis have disseminated tuberculosis, making the lymph node involvement a sign of systemic infection. Mann et al. [6] conducted a cohort study in a high-incidence and high-prevalence tuberculosis hospital in Rio de Janeiro between 2000 and 2016. They found a close relation between scrofuloderma, immunosuppression, and miliary tuberculosis [6,10].

Even though skin tuberculosis is caused by the same microorganism, the different types of cutaneous tuberculosis have different histologic findings. Endogenous types of skin tuberculosis (such as scrofuloderma) usually have granulomatous inflammation with caseous necrosis and acid-fast bacilli within the tissue. Inflammatory fistulae may have histologic findings of acute or chronic inflammation with central necrosis, as well as atrophic epidermis alongside scarring tissue. Other skin tuberculosis types, such as tuberculosis cutis miliaris, have non-specific histologic findings with necrotizing vasculitis and acid-fast bacilli [11].

To successfully diagnose cutaneous tuberculosis, clinicians must first perform a thorough clinical history and physical examination. A tuberculin skin test should be performed to confirm suspicion of other organ involvement. Once suspicion is raised, finding the organs involved in the infection must be the other task, and obtaining a biopsy of the affected tissue enables the culture and polymerase chain reaction test of the tissue sample. Histologic findings are not confirmatory of the diagnosis but suggest the diagnosis. Confirmatory diagnosis is with culture or a positive polymerase chain reaction, and if not available, the response to treatment can be used as a diagnostic criterion in medical centers without proper diagnostic tools [11].

Treatment for either miliary or cutaneous tuberculosis consists of two parts, i.e., intensive and maintenance. Intensive period comprises the daily administration of rifampin, isoniazid, pyrazinamide, ethambutol, and streptomycin for eight weeks, followed by the maintenance, using a daily dose of isoniazid and rifampin for 16 weeks [12].

## Conclusions

Recognizing skin infections caused by *M. tuberculosis* is crucial, as it disproportionately affects vulnerable populations, particularly children in impoverished regions such as Africa, India, and other underserved areas worldwide. Many of these individuals lack access to adequate healthcare due to systemic deficiencies. As healthcare professionals, we may encounter patients with severe limitations, including poor health and minimal resources. It is our responsibility to restore hope and provide them with the best possible care. Early identification of tuberculosis through skin lesions can be lifesaving, preventing multi-organ involvement and potentially fatal outcomes. A thorough physical examination, with a keen eye for cutaneous manifestations of *M. tuberculosis*, is essential for timely diagnosis and intervention.
